# Cultivation of *Caenorhabditis elegans* on new cheap monoxenic media without peptone

**DOI:** 10.21307/jofnem-2021-036

**Published:** 2021-04-01

**Authors:** Tho Son Le, T. T. Hang Nguyen, Bui Thi Mai Huong, H. Gam Nguyen, B. Hong Ha, Van Sang Nguyen, Minh Hung Nguyen, Huy-Hoang Nguyen, John Wang

**Affiliations:** 1 College of Forestry Biotechnology, Vietnam National University of Forestry, Hanoi, Vietnam; 2 Faculty of Biology, VNU University of Science, Vietnam National University, Hanoi, Vietnam; 3 Center for Molecular Biology, Institute of Research and Development, Duy Tan University, Da Nang, Vietnam; 4 Institute of Genome Research, Vietnam Academy of Science and Technology, Hanoi, Vietnam; 5 Biodiversity Research Center, Academia Sinica, Taipei, Taiwan.

**Keywords:** Growth media, Worm cultivation, *Caenorhabditis*, Brood size, Lifespan

## Abstract

The study of species biodiversity within the *Caenorhabditis* genus of nematodes would be facilitated by the isolation of as many species as possible. So far, over 50 species have been found, usually associated with decaying vegetation or soil samples, with many from Africa, South America and Southeast Asia. Scientists based in these regions can contribute to *Caenorhabditis* sampling and their proximity would allow intensive sampling, which would be useful for understanding the natural history of these species. However, severely limited research budgets are often a constraint for these local scientists. In this study, we aimed to find a more economical, alternative growth media to rear *Caenorhabditis* and related species. We tested 25 media permutations using cheaper substitutes for the reagents found in the standard nematode growth media (NGM) and found three media combinations that performed comparably to NGM with respect to the reproduction and longevity of *C. elegans*. These new media should facilitate the isolation and characterization of *Caenorhabditis* and other free-living nematodes for the researchers in the poorer regions such as Africa, South America, and Southeast Asia where nematode diversity appears high.

The nematode *Caenorhabditis elegans* is a lab workhorse and one of the best studied animals at the molecular and genetic levels. While *C. elegans* can be found in rotting fruits and leaves (Barriere and Felix, 2006; Felix and Braendle, 2010; Felix et al., 2013; Kiontke et al., 2011; Petersen et al., 2014; Samuel et al., 2016; Schulenburg and Felix, 2017), its distribution and ecology are poorly understood (Frezal and Felix, 2015; Kiontke et al., 2011). Even less natural history is known for other *Caenorhabditis* species, although many are also found in rotting vegetation (Felix et al., 2013; Ferrari et al., 2017; Stevens et al., 2019) while a few are associated with specific hosts (e.g., *C. inopinata*, *C. drosophilae, C. angaria*, and *C. bovis*) (Kanzaki et al., 2018; Kiontke, 1997; Stevens et al., 2020; Sudhaus et al., 2011).

Recently, there has been increased interest in biodiversity sampling of free-living nematodes, especially those in the *Caenorhabditis* (Crombie et al., 2019; Dolgin et al., 2008; Felix et al., 2014; Kiontke et al., 2011; Schulenburg and Felix, 2017; Stevens et al., 2019) and *Pristionchus* genera (Felix et al., 2018; Herrmann et al., 2019; Kanzaki et al., 2012, 2013, 2014; Susoy et al., 2016; Yoshida et al., 2018). Many free-living soil nematodes can be isolated by plating samples, such as soil or rotting vegetation, onto nematode growth media (NGM) agar plates seeded with bacteria, usually *Escherichia coli* strain OP50 that serves as food for the nematodes. Using this method, over 50 new *Caenorhabditis* species have been found, many in tropical or subtropical locations in Africa, South America, and Southeast Asia (Caenorhabditis Evolution Community, 2020) and often with very little effort. Thus, many new species await to be found.

The majority of nematode sampling effort has been done by researchers based or trained in Europe, Japan, or the USA. However, logistically local African, South American, and Southeast Asian scientists are better situated for sampling. Unfortunately, many scientists in these regions are often constrained by limited research resources.

Even the material costs, which can vary from country to country, could accumulate prohibitively for NGM agar plates, a defined medium composed of some salts, cholesterol, peptone, and agar (Brenner, 1974; Stiernagle, 2006). The salts (trace minerals) and cholesterol are directly essential for *C. elegans* survival and development (Flavel et al., 2018; Kawasaki et al., 2013; Matyash et al., 2001) while peptone is indirectly important by providing nutrients for bacterial growth. Agar serves as a solidifying agent (Stiernagle, 2006).

Inspired an earlier publication that used horse liver extract in axenic media (Dougherty, 1953) for rearing nematodes, we sought to find cheaper, crude extract replacements for cholesterol, peptone, and minerals. In this study, we tested 25 alternative media permutations for rearing *C. elegans*. Of these we found three media conditions that perform comparably to NGM with respect to reproductive output and longevity, and therefore may serve as an economical substitute to NGM.

## Materials and methods

### Worm strains and general methods

*C. elegans* Bristol var N2; *C. tropicalis* (BRC20400, a wild isolate from Taiwan); *C. briggsae* (CFB206, a wild isolate from Vietnam collected in this study). Worm strains were grown on NGM plates seeded with *Escherichia coli* OP50 and cultured in a 19 ± 1°C incubator, except where noted. Counting was conducted at room temperature. OP50 was cultured in a modified Luria-Bertani (LB) Broth (below) at room temperature (25–30°C) overnight prior to seeding the plates.

### Chemicals, solutions, and media

NaCl (Bio Basic Canada Inc., 7647-14-5), peptone (TM MEDIA, 1506), agar (TM MEDIA, 242 M), nutrient agar (TM MEDIA, TM341), plant cooking oil (Tuong An Company, Ngon), yeast extract (Bio Basic Canada Inc., G0961), cholesterol powder (Across Organics, 110190250), CaCl_2_ (Fisher, 10043-52-4), MgSO_4_ (Fisher, 10034-99-8), KH_2_PO_4_ (Merck, 7778-77-0), and K_2_HPO_4_ (Fisher, 7758-11-4). All chemicals were stored at room temperature (25–30°C).

### Mineral mixture

1 mL of 1 M CaCl_2_, 1 mL of 1 M MgSO_4_, and 25 mL of 1 M KPO_4_ (pH 5.57) were mixed together in 1 L of double distilled water and then autoclaved at 118°C for 20 min.

### Market products

Straw mushrooms (*Volvariella volvacea*), oyster mushrooms (*Pleurotus ostreatus*), pig fat, and chicken eggs were bought from the outdoor Xuan Mai Market, Xuan Mai, Hanoi, Vietnam.

### Mushroom solution

For *V. volvacea*, we tested two stages, “tiny button” and “egg”, while for *P. ostreatus* we used only the “fruiting” stage. For each sample, 200 g was ground with a mortar and a pestle in 500 mL of distilled water and then filtered with a fine mesh strainer. To prevent possible loss of heat labile nutrients, half of the raw mushroom solution was not autoclaved and directly frozen at −10°C; solutions for the two mushroom species were frozen separately. The remaining raw mushroom solutions of both species was combined and then autoclaved to sterilize against any bacterial or fungal contamination.

### Soil solution

An approximately 1,000 g soil sample was collected underneath grass from a field in Cao Phong District, Hoa Binh Province, Vietnam (20^o^44’18.6” N, 105^o^19’003” E). A grassy site was chosen because such a site presumably contained more minerals, and perhaps other nutrients, than sites without vegetation. Digging of soil started at a depth of ~10 cm covering a ~40 cm radius and continued downward for another ~5 cm. A portion of this soil (125 g) was mixed into 470 mL of distilled water, distributed into ten 50-ml tubes, and then centrifuged with an Eppendorf Centrifuge 5810 R using a 50-ml rotor at 2,000 rpm for 5 min. The upper solution was decanted into a new flask and the sediment pellet was discarded.

### Egg mixture

One whole egg was manually whipped together with a stirring rod for about 5 to 10 min until the egg white and yolk were completely mixed based on appearance. This mixture was frozen as the stock egg mixture.

### Modified Luria-Bertani (LB) media

5 g of yeast extract, 1 g of nutrient agar, and 10 g of NaCl were mixed together in 1 L of distilled water, autoclaved at 118°C for 20 min, and then poured into Petri plates (Sambrook and Russell, 2001).

### Plain agar

17 g of agar and 3 g of NaCl were mixed together in 1 L of distilled water, autoclaved at 118°C for 20 min, and then poured into Petri plates.

### Preparation of alternative media

We made 25 different media types (Nematode Cheap Media; NcM1 to NcM25) using the media components listed in Table 1. Except for media with “unautoclaved” mushroom solution, we mixed all components together and autoclaved them at 118°C for 20 min in 250 mL volumes. For media with “unautoclaved” mushroom, we first autoclaved 245 mL of all the components minus the mushroom solution and then added 2.5 mL of each of the two mushroom solutions (i.e., 5 mL total) to the post-autoclaved media while it was still warm. NGM was prepared following the standard protocol (Stiernagle, 2006). All media were poured into glass Petri plates, allowed to solidify, and then seeded with an overnight culture of *E. coli* OP50.

**Table 1.** Media components of NcMs and NGM.

							Source of cholesterol^a^																		
	Plant cooking oil						Chicken egg						Pig fat						Mushroom						Soil
Component (Amount/ L)	NcM	NcM	NcM	NcM	NcM	NcM	NcM	NcM	NcM	NcM	NcM	NcM	NcM	NcM	NcM	NcM	NcM	NcM	NcM	NcM	NcM	NcM	NcM	NcM	NcM
**Plant cooking oil** (100 µL)	Ѵ	Ѵ	Ѵ	Ѵ	Ѵ	Ѵ																			
**Chicken egg** (0.56 g)							Ѵ	Ѵ	Ѵ	Ѵ	Ѵ	Ѵ													
**Pig fat** (0.4 g)													Ѵ	Ѵ	Ѵ	Ѵ	Ѵ	Ѵ							
**Autoclaved mushroom solution** (20 mL)		Ѵ		Ѵ		Ѵ		Ѵ		Ѵ		Ѵ		Ѵ		Ѵ		Ѵ	Ѵ	Ѵ	Ѵ				
**Non-autoclaved mushroom solution** (20 mL)																						Ѵ	Ѵ	Ѵ	
**Pure mineral mixture** (1 mL of 1 M CaCl_2_, 1 mL of 1 M MgSO_4_, 25 mL of 1 M KPO_4_)	Ѵ	Ѵ					Ѵ	Ѵ					Ѵ	Ѵ					Ѵ			Ѵ			
**Soil solution** ^**b**^(10 mL)					Ѵ	Ѵ					Ѵ	Ѵ					Ѵ	Ѵ			Ѵ			Ѵ	Ѵ
**Agar** (17 g)	Ѵ	Ѵ	Ѵ	Ѵ	Ѵ	Ѵ	Ѵ	Ѵ	Ѵ	Ѵ	Ѵ	Ѵ	Ѵ	Ѵ	Ѵ	Ѵ	Ѵ	Ѵ	Ѵ	Ѵ	Ѵ	Ѵ	Ѵ	Ѵ	Ѵ
**NaCl** (4 mL of 0.75 g/ mL)	Ѵ	Ѵ			Ѵ	Ѵ	Ѵ	Ѵ	Ѵ	Ѵ	Ѵ	Ѵ	Ѵ	Ѵ	Ѵ	Ѵ	Ѵ	Ѵ	Ѵ	Ѵ	Ѵ	Ѵ	Ѵ	Ѵ	Ѵ
Media type	NcM	NcM	NcM	NcM	NcM	NcM	NcM	NcM	NcM	NcM	NcM	NcM	NcM	NcM	NcM	NcM	NcM	NcM	NcM	NcM	NcM	NcM	NcM	NcM	NcM
	1	2	3	4	5	6	7	8	9	10	11	12	13	14	15	16	17	18	19	20	21	22	23	24	25

Notes: ^a^Pure cholesterol was replaced with chicken egg, pig fat and/or mushroom extract. Mushroom may have nutrients in addition to cholesterol. ^b^The soil solution was intended as a replacement for the pure minerals (salt mixture) in NGM, however it may have included additional nutrients.

### Brood size assays

Prior to the brood assays, L1 stage worms growing on NGM were transferred onto the test media to allow acclimation for one generation. From their progeny, a batch of 2 to 10 (mode = 7) synchronized L2 hermaphrodites (P0) were placed onto each bacterium-seeded test media plate. The P0s were transferred to a new plate every day until they stopped laying eggs. Eggs on each plate were allowed to hatch and develop until the L2 to L4 stages. Then, to facilitate counting, the F1 offspring were killed with mild heat, which preserves the worm’s shape, by passing the plates over the flame of an alcohol lamp as follows. The plates were held barehanded with the open agar surface facing the flame about one to two inches away; the plates were moved back and forth for up to about 10 seconds until the plates felt a little warm.

Average brood sizes were first determined per batch of P0s (total brood across all days divided by the initial number of P0s). Then, the global average was determined by averaging the per batch average brood sizes. Note, the standard errors (SE) for each data set is the SE among P0 batches and not the SE across individual P0s. In addition, a batch smooths out the variation among individuals. Thus, among the batch SE is expected to be smaller than among individual SEs. Brood size comparisons were analyzed with one-way ANOVA and then Dunnett’s post-hoc test.

### Lifespan assay

Ten L1 worms (P0) that were growing on standard NGM media were transferred to a test media plate and allowed to grow and reproduce offspring. After, 10 to 50 newly hatched L1 F1s were transferred to a fresh test media plate to start the lifespan assay. These worms were transferred to a new media plate every two or three days. A worm was scored as dead if its body was still present on the agar surface of the plate and it did not move when tapped with a worm pick. A few worms that died on the edge of the plate were not counted (censored) in the lifespan data set. Lifespan was analyzed using the log-rank (Mantel-Cox) test (code: Pairwise_survdiff(Surv(Longevity, Stage code) ~ Media, p.adjust = ”bonferroni”, data = data set) to compare each NcM with NGM (Kassambara, 2020).

### Media preference assay

Worms that had been growing on one of seven “source” NcMs (NcM6, NcM8, NcM9, NcM12, NcM18, NcM20, and NcM25) were tested if they preferentially migrated to one of the same seven NcMs or NGM “destination plugs”. For these assays, all source media plates were seeded within a 15 min time window with 90 µL of an overnight *E. coli* OP50 culture, and then incubated for five days at room temperature prior to placing worms onto the plates. Then, five to seven P0 L2 larvae were placed on each of the seven OP50-seeded source media plates (NcM6, NcM8, NcM9, NcM12, NcM18, NcM20 and NcM25) and allowed to grow and proliferate for two generations (F1 and F2) before testing on the media preference test assay plates.

**Figure 1:**
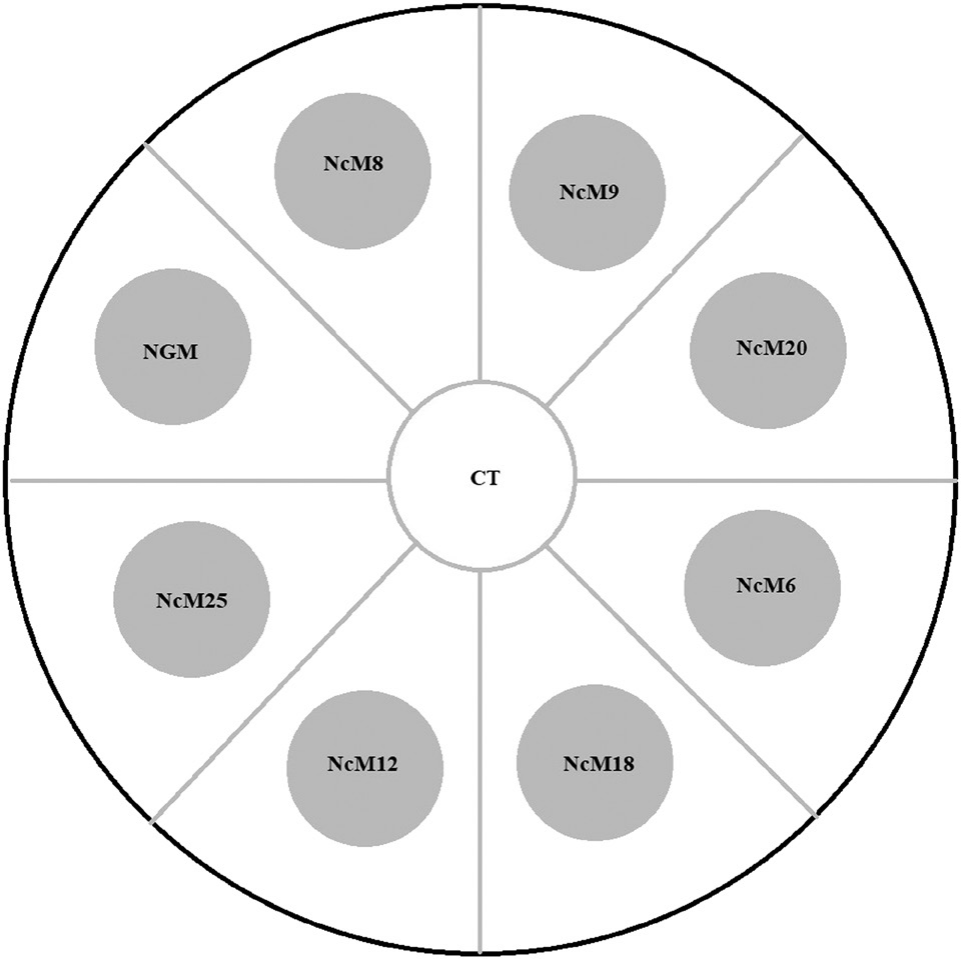
Schematic of media preference assay. OP50-seeded plugs of the eight indicated NcMs (grey circles) are placed onto a 9 cm-diameter plain agar plate 1 h before the preference assay. At the start of the assay, 30 well-fed young adult F2 worms are placed within the center circle (CT) and then allowed to migrate. After 2 h, the locations of the worms were recorded.

Test assay plates were plain agar plates (no nutrients and no OP50, see above) onto which one 1.7-cm diameter plug (cut using a standard test tube) from each of the eight new OP50-seeded destination plates (seven NcM destination media and NGM) was placed; plugs were evenly spaced and placed radially 1.0 cm from the center (Fig. 1). After letting the assembled test assay plate “settle” for one hour at room temperature, 30 young-adult F2 worms were placed in the center of a test plate. The worms could then freely migrate. After two hours, the locations of the worms were recorded (on each plug, in the center, or not observed [called “Lost”]). The preference tests were conducted in the winter time so room temperature was around 16–18°C. Four biological replicates per source media type were conducted for a total of 28 assays.

To ascertain whether there would be any relationship between a particular media source and media preference levels, and a group of media destinations and media preference levels, the effects of either each media source or destination were analyzed using a generalized linear model (GLM; code: summary(glht(aov,linfct=mcp(in=”Dunnett”)))) (Hothorn et al., 2008). Dunnett’s post-hoc test was used to compare each preference percentage against one of two destinations: the source-as-destination (i.e., when both source and destination were the same, e.g., both NcM6) or NGM.

### Estimation of relative OP50 growth supported by different NcM plates

For each of the seven media used in the preference assay and NGM, 6 mL of the growth media was poured into 6-cm Petri plates and allowed to solidify. Next, 50 µL of an OP50 suspension was spread onto each plate and then the plates were incubated at 25–30°C for three days. After, the OP50 lawn on each plate was washed off by adding 1 mL of distilled water and shaking the plate on a shaking machine at 80 rpm for 15 min; this was repeated once and the two suspensions were pooled. The OD_600_ value was then measured on a 752B VIS spectrophotometer. Three replicates of each growth media were tested; replicates were done in parallel. To minimize environmental differences, all media were poured on the same day and all experiments were conducted in parallel.

### Isolation wild nematodes on four NcMs

Nematodes found in rotting vegetation were isolated following standard methods (Barriere and Felix, 2006). Sixteen rotting vegetation samples were collected during the third week of November 2020 in Cat Tien National Park in Vietnam. Approximately 20–30 g of each sample was placed on OP50-seeded NcM6, NcM8, NcM12, NcM18, and NGM plates in parallel (9-cm diameter) and incubated for 3 to 7 days at room temperature. All sixteen samples yielded nematodes on at least one of the five media plates. Only the sample for which nematodes were first observed (11^o^23’55.9” N, 107^o^20’15.3” E; on day 3) is reported in detail because, unfortunately, the nematodes from the other 15 samples were lost due to technical reasons.

For this sample, more than one type of nematode was observed on the NcM18 plates. The adult nematodes that had similar features (body size and shape of head and tail) to *C. elegans* were individually picked onto different plates and allowed to reproduce. Next, 10 individuals of each strain were observed under a dissecting microscope more carefully. The worms with two circular pharyngeal bulbs were candidates for *Caenorhabditis* species and five such adults were pooled together and lysed using the single worm PCR protocol, subjected to 18 S rDNA PCR using the SSU18A (5’–AAAGATTAAGCCATGCATG–3’) and SSU26R (5’–CATTCTTGGCAAATGCTTTCG–3’) primers (Barriere and Felix, 2006), and then sequenced. The sequence was aligned to the nucleotide database by nucleotide BLAST on the National Center for Biotechnology Information (NCBI) website (Zhang et al., 2000). This *C. briggsae* isolate was designated CFB206.

### *Isolation of* Acinetobacter *sp. strain CFBb9*

The same sample used to isolate nematodes, above, was used to isolate wild bacterial strains. Approximately 17 g of rotting vegetation was put in a 15 mL tube filled with 10 mL of sterile water and then mixed by pipetting up and down. This first solution was further diluted 1,000-fold in sterile water and then 100 µL of the diluted solution was spread onto a LB-agar plate (9-cm diameter). The plate was incubated at room temperature (20–25°C for these experiments) for 2 days. Two bacterial colonies grew on the plate and each was individually picked into LB broth, cultured at room temperature for DNA isolation, and frozen in 25% (v/v) glycerol. One bacterial strain was identified based on 16 S rDNA PCR amplification using the QUGP-Fn5 (5’–ACTCCTACGGGAGGCAGCAG–3’) and QUGP-Rn2 (5–TGACGGGCGGTGTGTACAG–3’) primers (Vingataramin and Frost, 2015) followed by sequencing and nucleotide BLAST comparison to the NCBI nucleotide database. This *Acinetobacter* isolate was designated CFBb9. The other isolate was not analyzed and stored for future research.

### General statistics

All statistical analyses were conducted in R Software (versions 3.63 and 4.0.4 (R Core Team, 2017)). The single step Bonferroni correction method was used to control for multiple test correction.

## Results

We reasoned that several of the reagents in NGM could potentially be replaced by cheaper, less well-defined components and still support robust *C. elegans* growth. For cholesterol, we considered three common products: eggs, pig fat, and a mixture of straw and oyster mushrooms. In addition, vegetable cooking oil has no cholesterol and protein but it has high amounts of lipids, vitamin A, and E. The eggs and mushrooms also have high amounts of protein, so they could potentially serve as a replacement for peptone. Finally, we made a soil solution with the intention of replacing the minerals in NGM, however, this soil solution may have had additional nutrients, possibly because it was sampled under grass.

We combined these five components along with subsets of the standard NGM reagents in 25 media permutations (or Nematode Cheap Media, NcM, Table 1). We first confirmed that all 25 NcMs could support OP50 growth. Lawns were observed on the OP50-seeded plates even on the presumptively most nutrient-poor media (NcM25: soil + agar + NaCl); this was not contamination as control media plates without seeding OP50 did not exhibit any bacterial growth. Next, we conducted two assays to examine how well *C. elegans* grew on the NcMs as compared to NGM.

### Brood sizes on OP50-seeded NcMs

**Figure 2:**
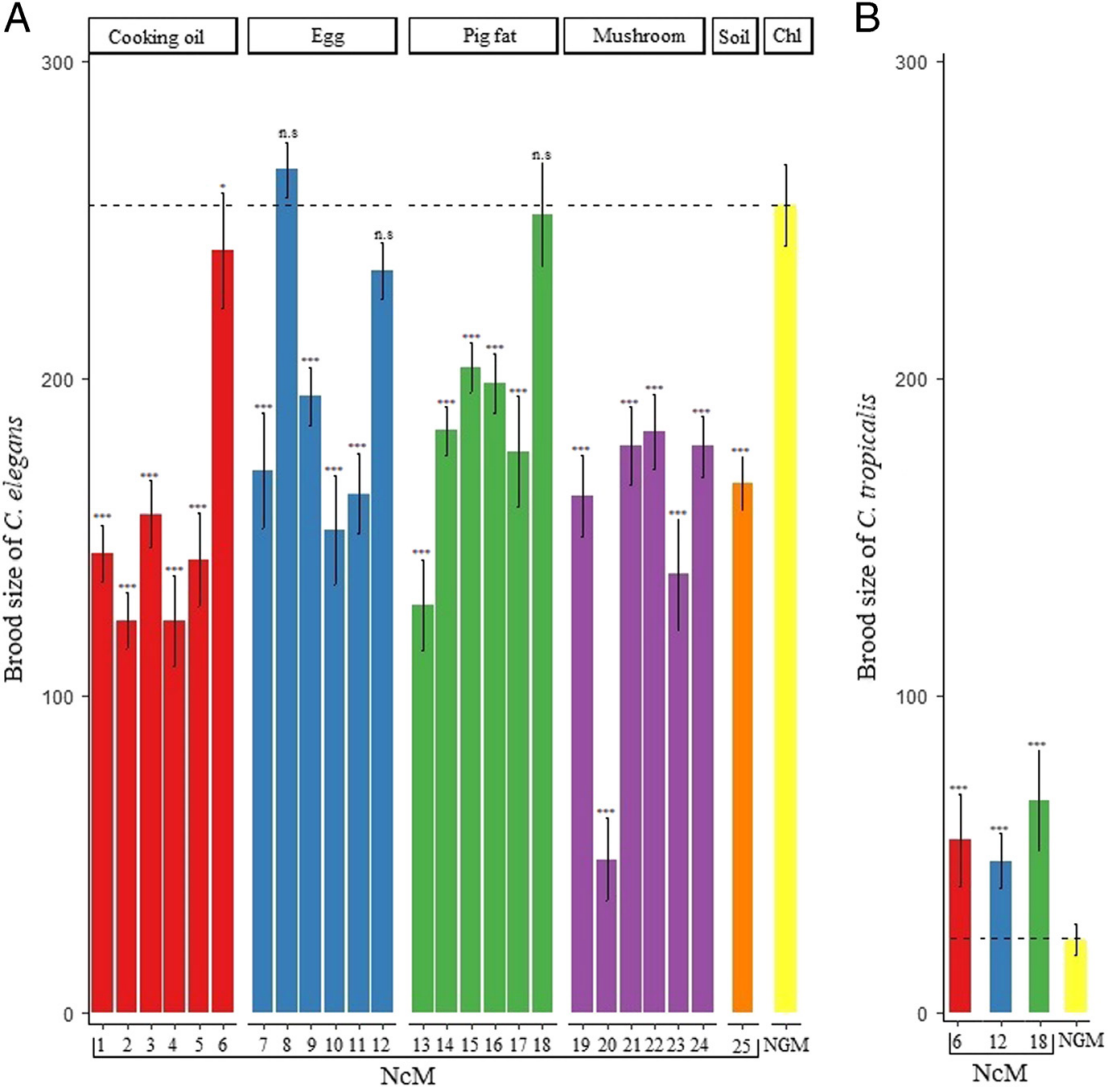
Brood sizes of *C. elegans* and *C. tropicalis* on test media and the NGM control. (A) The total self-brood sizes of *C. elegans* are presented as means ± 1 SE. Brood sizes differed among the media (*P* < 2e−16, F = 97.0, one-way ANOVA test). The Dunnett’s post-hoc test was used for brood-size comparisons of the test media against the NGM control (^***^ *P* < 0.001; n.s, not significant, i.e., *P*  > 0.05), see Table S1 for exact *P-*values. Chl, cholesterol. (B) The total self-brood sizes of *C. tropicalis* differed among the 3 NcMs and NGM (*P* = 6.3e−10, F = 17.9, one-way ANOVA test). The Dunnett’s post-hoc test is used for brood-size comparisons of the media against the NGM control (^***^ *P* < **0.001; *P < **0.5), see Table S2 for exact *P*-values. Note: The standard errors (SE) and error bars for each data set are the SE among P0 batches and not the SE across individual P0s.

The ability for *C. elegans* to proliferate is the most important criterion of the alternative growth media. Thus, in the first test, we determined the hermaphrodite brood sizes on each media. For three media combinations (NcM8, NcM12, and NcM18), we obtained mean numbers of offspring (all means ≥ 234.0), which were not significantly different from that of NGM (254.7 mean ± 12.8 standard error (SE); all *P* > 0.05; one-way ANOVA followed by Dunnett’s test for difference from NGM; Fig. 2A and Table S1). All three of these NcMs had a “complete” nutrient complement: cholesterol (from egg or pig fat), protein (peptone, mushrooms, or eggs), and minerals (salt mixture or soil solution). The other 22 NcM combinations had significantly fewer offspring than NGM (all means < 240.5; all *P* < 0.05, one-way ANOVA followed by Dunnett’s test for difference from NGM; Fig. 2A and Table S1). Altogether, these results suggested that three NcMs could be potential alternatives for rearing *C. elegans*.

**Table S1.** Brood sizes of *C. elegans* on 25 test media and the NGM control.

Media	Number of tested hermaphrodites	Total brood size (Mean ± SE)	Dunnett’s post-hoc *P-*value
NGM	39	254.7 ± 12.8	Control
NcM1	57	144.9 ± 8.8	<0.001
NcM2	50	123.8 ± 8.7	<0.001
NcM3	55	157.5 ± 10.6	<0.001
NcM4	55	123.8 ± 14.4	<0.001
NcM5	25	142.9 ± 14.8	<0.001
NcM6	42	240.5 ± 18.0	0.0228
NcM7	17	171.1 ± 18.3	<0.001
NcM8	26	265.8 ± 8.7	0.5468
NcM9	32	194.5 ± 9.1	<0.001
NcM10	29	152.3 ± 17.3	<0.001
NcM11	24	163.9 ± 12.5	<0.001
NcM12	21	234.0 ± 8.8	0.1817
NcM13	50	128.9 ± 14.2	<0.001
NcM14	47	183.6 ± 7.5	<0.001
NcM15	43	203.6 ± 7.9	<0.001
NcM16	40	198.6 ± 9.3	<0.001
NcM17	38	177.2 ± 17.5	<0.001
NcM18	44	251.7 ± 16.2	1.0
NcM19	38	163.2 ± 12.9	<0.001
NcM20	35	48.7 ± 12.9	<0.001
NcM21	26	178.9 ± 12.4	<0.001
NcM22	35	183.4 ± 11.6	<0.001
NcM23	16	138.4 ± 17.3	<0.001
NcM24	39	178.7 ± 9.5	<0.001
NcM25	45	167.0 ± 8.2	<0.001

Note: Gray shading highlights the three media on which worms had brood sizes not different from NGM. Pink shading highlights NcM6 which was only slightly inferior to NGM. Worms on all the remaining NcM media yielded fewer worms than NGM control (yellow). The standard errors (SE) and error bars for each data set are the SE among P0 batches and not the SE across individual P0s.

**Table S2.** Brood sizes of *C. tropicalis* on different media and the NGM control.

Media	Number of tested hermaphrodites	Total brood size (Mean ± SE)	Dunnett’s post hoc *P-*values
NGM	37	23.3 ± 5.0	Control
NcM6	42	54.8 ± 14.6	<0.001
NcM12	37	48.2 ± 8.4	<0.001
NcM18	32	67.2 ± 15.7	<0.001

Note: Worms had bigger brood sizes on all three NcMs than NGM control (yellow). Note, the standard errors (SE) and error bars for each data set are the SE among P0 batches and not the SE across individual P0s.

Next, to examine the broader potential of NcMs for *Caenorhabditis* species, we tested the brood production of another hermaphroditic species, *C. tropicalis*, on two of the three “best” NcM candidates (NcM12 and NcM18) and NcM6 (because it supported nearly as many progeny as the three best for *C. elegans*), in the same fashion. We found that the mean numbers of offspring on all three NcMs (all means between 48.6 and 67.2) were significantly higher than that on NGM (23.3 mean ± 5.0 SE; all *P* < 0.05, one-way ANOVA followed by Dunnett’s test for difference from NGM; Fig. 2B Table S2). Note, *C. tropicalis* brood sizes were lower than for *C. elegans* (all means between 234.0 and 251.7; all *P* < 2.2e−16, Welch Two Sample *t*-test). This result indicates that these three NcMs, and especially NcM18, could be used to isolate *C. tropicalis* and by extension, probably many other *Caenorhabditis* nematodes. Furthermore, this result suggests that NGM is incomplete, at least for *C. tropicalis*.

### Longevity on OP50-seeded NcMs

Longevity is another indicator of the growth media quality. To evaluate the effects of the media on the lifespan of *C. elegans* in comparison with NGM, we tested seven media. We chose the three media supporting brood sizes comparable to NGM (NcM8, NcM12, and NcM18) as well as NcM6 and NcM9 because they supported nearly as many progeny as the previous three media (see Table S1). We also chose two additional media, NcM20 because it was the poorest performing media and NcM25 because it was presumptively of lowest nutrient quality since it lacked obvious cholesterol and protein.

**Figure 3:**
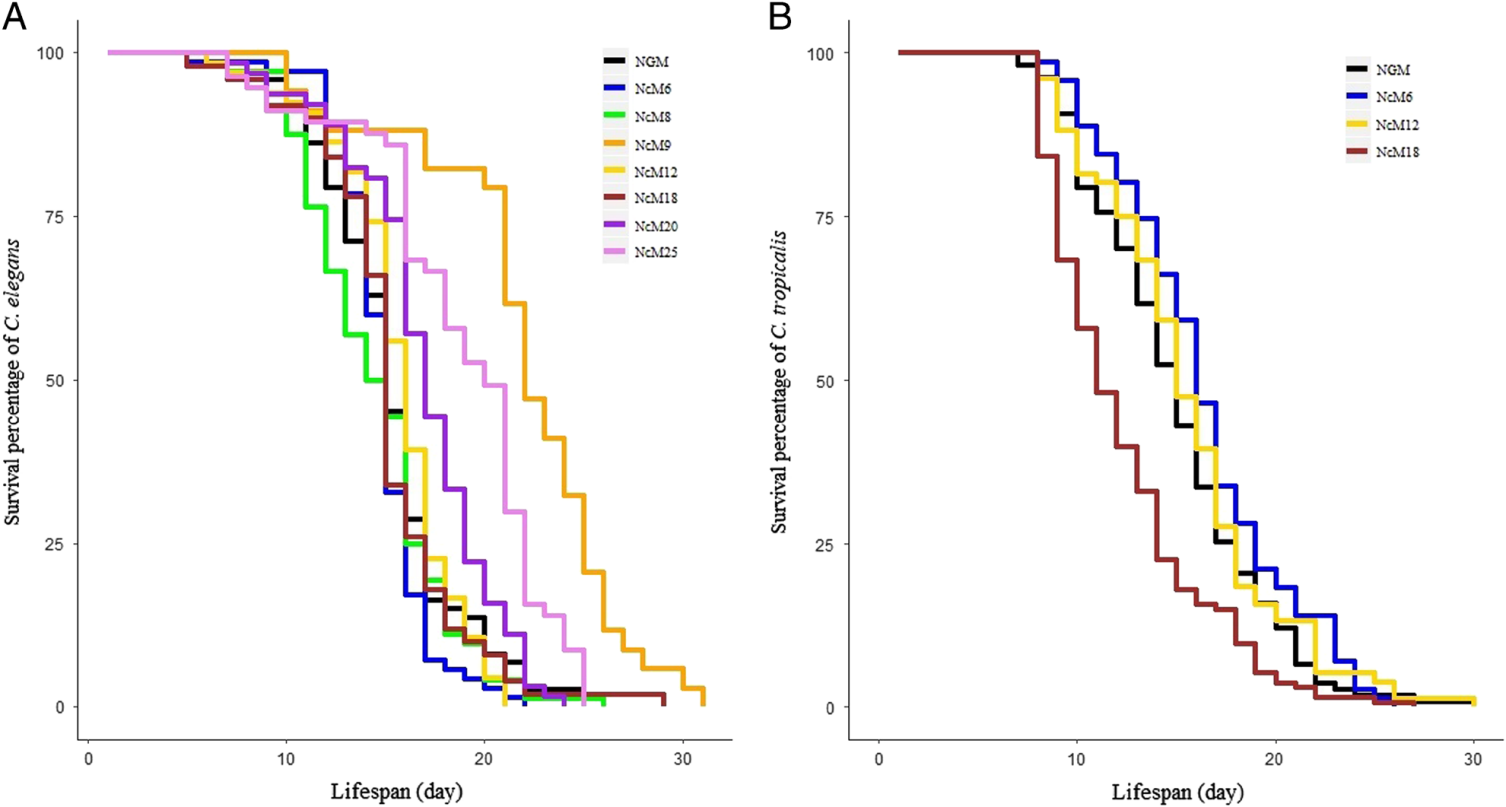
Lifespan assays of *C. elegans* and *C. tropicalis* on different media and the NGM control. (A) *C. elegans* worms grown on NcM9, NcM20, and NcM25 survived longer than on the NGM control (all *P* ≤ **0.01, log-rank test), while worms grown on NcM6, NcM8, NcM12, and NcM18 had similar lifespans as the control (*P* ≥ **0.1, log-rank test). Detailed lifespan data are in Tables S3 and S4. (B) *C. tropicalis* worms grown on NcM6 and NcM12 have similar lifespans as the NGM control (both *P* > **0.05, log-rank test), while worms grown on NcM18 have shorter lifespans than the control (*P* < **2e−5, log-rank test), see Tables S5 and S6.

We found that worms growing on the media with the highest brood counts (NcM6, NcM8, NcM12, and NcM18) and NcM20 had lifespans (all means ≥ 14.5 days) not significantly different from NGM (15.2 ± 0.4, mean ± 1 SE; all *P*-values > 0.05, log-rank test; Fig. 3A and Tables S3 and S4). In contrast, worms had longer average lifespans growing on the two other NcMs (NcM9 and NcM25) than on NGM (all *P-*values < 0.01; Fig. 3A and Tables S3 and S4). While the worms did grow on NcM25 for this assay (which used F1 worms, see Methods), it should be noted that this media lacks a cholesterol source, and large numbers of F3 generation worms died upon continued passage of worms to new plates.

**Table S3.** Lifespan assays of *C. elegans* on 8 different media.

Media	Number of tested hermaphrodites	Lifespan (days) Mean ± SE	Log-rank *P-*value
NGM	73	15.2 ± 0.4	Control
NcM6	70	14.8 ± 0.3	1.0
NcM8	72	14.5 ± 0.4	1.0
NcM9	34	21.9 ± 0.9	4e−09
NcM12	66	15.6 ± 0.4	1.0
NcM18	50	15.1 ± 0.5	1.0
NcM20	63	16.9 ± 0.5	0.39
NcM25	57	18.8 ± 0.6	2.4e−05

Note: Gray shading highlights the media on which worms had longer lifespans than NGM control (red).

**Table S4.** Number of surviving *C. elegans* worms on 8 different media at each day of the lifespan assays.

	Number of surviving worms							
Day	NGM	NcM6	NcM8	NcM9	NcM12	NcM18	NcM20	NcM25
1	73	70	72	34	66	50	63	57
2	73	70	72	34	66	50	63	57
3	73	70	72	34	66	50	63	57
4	73	70	72	34	66	50	63	57
5	72	69	72	34	66	49	63	57
6	72	69	72	34	65	49	63	57
7	70	69	70	34	64	48	62	55
8	70	69	70	34	64	48	61	54
9	70	68	70	34	62	46	59	52
10	67	68	63	32	61	46	59	52
11	63	68	55	31	60	45	58	51
12	58	62	48	30	57	42	56	51
13	52	55	41	30	54	39	52	51
14	46	42	36	30	49	33	51	50
15	33	23	32	30	37	17	47	49
16	21	12	18	30	26	13	36	39
17	12	5	14	28	15	9	28	38
18	11	4	8	28	11	6	21	33
19	10	3	7	28	7	5	14	30
20	6	2	3	27	3	4	10	28
21	5	1	3	21	0	2	7	17
22	2	0	1	16		1	2	9
23	2		1	14		1	1	8
24	2		1	11		1	0	5
25	1		1	7		1		0
26	0		0	4		1		
27				3		1		
28				2		1		
29				2		0		
30				1				
31				0				
Censored	7	10	8	6	14	5	17	23

**Table S5.** Lifespan of *C. tropicalis* on 4 different media.

Media	Number of tested hermaphrodites	Lifespan (days) Mean ± SE	Log-rank *P*-value
NGM	107	14.96 ± 0.42	Control
NcM6	71	16.35 ± 0.51	0.4
NcM12	76	15.49 ± 0.53	1.0
NcM18	133	12.3 ± 0.35	1.3e−05

Note: Gray shading highlights the media on which worms had lifespans not different from NGM control (red).

**Table S6.** Number of surviving *C. tropicalis* worms on 4 different media at each day of the lifespan assays.

	Number of surviving worms			
Day	NGM	NcM6	NcM12	NcM18
1	107	71	76	133
2	107	71	76	133
3	107	71	76	133
4	107	71	76	133
5	107	71	76	133
6	107	71	76	133
7	105	71	76	133
8	103	70	73	112
9	97	68	67	91
10	85	63	62	77
11	81	60	61	64
12	75	57	57	53
13	66	53	52	44
14	56	47	45	30
15	46	42	36	24
16	36	33	30	21
17	27	24	21	20
17	22	20	14	13
19	17	15	12	7
20	13	13	10	5
21	7	10	10	4
22	4	10	4	2
23	3	5	4	2
24	2	2	4	2
25	2	1	3	1
26	2	0	1	1
27	1		1	0
28	1		1	
29	1		1	
30	0		0	

Next, we similarly determined the longevity of *C. tropicalis* on two of the three “best” NcMs (NcM12 and NcM18) and NcM6. The lifespans on NcM6 and NcM12 (16.35 and 15.49 days, respectively) were not significantly different from that on NGM (14.96 ± 0.42, mean ± 1 SE, both *P-*values > 0.05, log-rank test; Fig. 3B and Tables S5 and S6), while that of NcM18 (12.3 ± 0.35) was significantly shorter (*P* ≤ 0.001, log-rank test; Fig. 3B and Tables S5 and S6).

### Correlation between number of OP50 cells and brood size and lifespan

Previous studies have shown that food amount correlated positively with brood size but negatively with lifespan (Lenaerts et al., 2008; Yu et al., 2015). We tested for a correlation between brood size and lifespan across the different NcMs and NGM for which both were assayed. Our brood size and lifespan results varied among the eight different media, and somewhat in opposite directions (rho = −0.76, *P* = 0.037, Spearman test). To examine if these two traits were also correlated with the amount of *E. coli* OP50 supported by the respective NcMs and NGM, we quantified OP50 cell number on these media and then conducted a correlational analysis. We found no significant correlation between OP50 amount and brood size (rho = 0.29, *P* = 0.5, Spearman test) or lifespan (rho = −0.48, *P* = 0.24, Spearman test). This suggests that some other factors in those NcMs could be involved in the variation of these two phenotypes.

### Media preference on OP50-seeded NcMs

Because we had multiple media sources available, we considered the possibility that *C. elegans* would tend to prefer the media on which they grew up (“source”) over new media they had never experienced in life. To do this, we focused on the seven media for which we had tested longevity (NcM6, NcM8, NcM9, NcM12, NcM18, NcM20, and NcM25). To ensure that worms were acclimated to the source media, we used young adult hermaphrodites whose ancestors had been raised on the source media for two generations (see Methods section). To conduct each assay, we placed 30 worms from one source media into the center of a test assay plate, let them migrate for 2 h, and then recorded their location (see Methods section).

**Figure 4:**
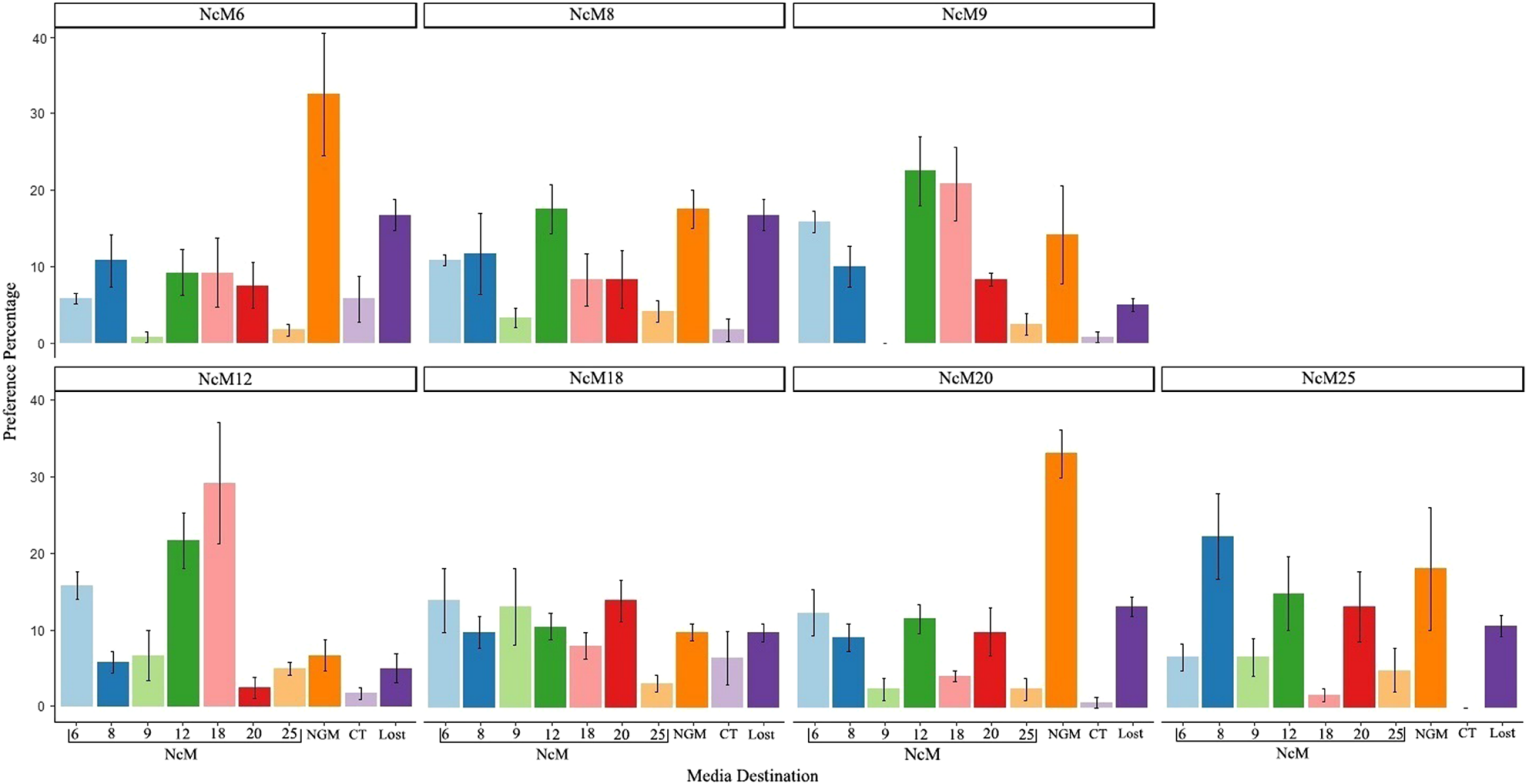
Media preference assays. Plots are the percentage of the worms located on the media destinations 2 hours after placing worms in the center of the test assay plates. GLM was used to analyze the correlation between the destinations and percentages of worms migrating to the destinations for all the seven-media sources (*P* = **1.0) and 10 destination-groups (*P* = **0.342, see more in Table S7). Dunnett’s post-hoc test was used to analyze *P*-values for each media destination group (in this figure) or each media destination in comparison with the source and NGM (Table S8). Names of media sources are labeled at the top of each subplot. X-axis, names of media destinations; CT, center; in every assay, a small number of tested worms are not observed (“Lost”).

Analysis of the data using a generalized linear model revealed that neither source nor destination media had a significant linear regression relationship with preference percentage (*P* = 1.0 for sources and *P* = 0.34 for destinations). However, when NcM9 or NcM12 were the source media, their destination preference patterns differed from the other sources (*P* < 0.05, Fig. 4 and Table S7), possibly indicating that growth experience on these two source media biases how the worms choose their destination. In addition, we examined if the destination media preference was the same as the source media, but this was not the case; instead, the migration pattern was complex (Fig. 4 and Table S8). Finally, we note that this migration assay may actually be measuring aversion to one or more of the NcMs including the source NcM.

**Table S7.** *P-*values of generalized linear model of each destination group and preference percentage.

Source	All	NcM6	NcM8	NcM9	NcM12	NcM18	NcM20	NcM25
*P*_(percentage~ destination)_	0.342	0.879	0.67	0.0205	5.84e−05	0.571	0.2198	0.1003

**Table S8.** *P-*values of the Dunnett’s test for media preference of *C. elegans*.

Source	NcM6			NcM8			NcM9			NcM12			NcM18			NcM20			NcM25		
Comparison Destination	mean ± SE	NcM6^a^	NGM^b^	mean ± SE	NcM8^a^	NGM^b^	mean ± SE	NcM9^a^	NGM^b^	mean ± SE	NcM12^a^	NGM^b^	mean ± SE	NcM18^a^	NGM^b^	mean ± SE	NcM20^a^	NGM^b^	mean ± SE	NcM25^a^	NGM^b^
NcM6	5.8 ± 0.7	Ref	<0.001	10.8 ± 0.7	1.0	0.6302	15.8 ± 1.4	0.02654	1.0	15.8 ± 1.8	0.84529	0.41764	14.1 ± 4.2	0.723	0.93	12.5 ± 3.0	0.9786	<1e−05	6.7 ± 1.7	1.0	0.3859
NcM8	10.8 ± 3.4	0.956	0.00641	11.7 ± 5.3	Ref	0.7559	10.0 ± 2.6	0.29024	0.9642	5.8 ± 1.4	0.03458	1.0	9.9 ± 2.1	1.0	1.0	9.2 ± 1.8	1.0	<1e−05	22.5 ± 5.6	0.0711	0.9923
NcM9	0.8 ± 0.7	0.0956	<0.001	3.3 ± 1.2	0.39	0.324	0.0 ± 0.0	Ref	0.0571	6.7 ± 3.3	0.05005	1.0	13.3 ± 5.0	0.845	0.98	2.5 ± 1.4	0.174	<1e−05	6.7 ± 2.4	1.0	0.3861
NcM12	9.2 ± 3.0	0.997	0.00139	17.5 ± 3.2	0.756	1.0	22.5 ± 4.5	<0.001	0.4818	21.7 ± 3.6	Ref	0.05008	10.7 ± 1.7	0.997	1.0	11.7 ± 1.9	0.9986	<1e−05	15.0 ± 4.8	0.5534	0.9984
NcM18	9.2 ± 4.5	0.997	0.00304	8.3 ± 3.4	0.984	0.2928	20.8 ± 4.8	0.00216	0.7125	29.2 ± 7.9	0.63337	0.00142	8.2 ± 1.8	Ref	1.0	4.2 ± 0.7	0.4101	<1e−05	1.7 ± 0.8	0.9984	0.0938
NcM20	7.5 ± 3.0	1.0	0.00300	8.3 ± 3.8	0.984	0.2929	8.3 ± 0.8	0.48166	0.821	2.5 ± 1.4	0.00731	0.97096	14.1 ± 2.7	0.731	0.935	10.0 ± 3.1	Ref	<1e−05	13.3 ± 4.6	0.7350	0.9756
NcM25	1.7 ± 0.8	0.985	<0.001	4.2 ± 1.4	0.505	0.0491	2.5 ± 1.4	0.99881	0.1598	5.0 ± 0.8	0.0239	0.99996	3.3 ± 1.1	0.855	0.61	2.5 ± 1.4	0.1741	<1e−05	5.0 ± 2.8	Ref	0.2531
NGM	32.5 ± 8.0	<0.001	Ref	17.5 ± 2.5	0.756	Ref	14.2 ± 6.4	0.05703	Ref	6.7 ± 2.0	0.0499	Ref	9.9 ± 1.1	1.0	Ref	33.3 ± 3.1	<0.001	Ref	18.3 ± 8.0	0.2531	Ref
Center	5.8 ± 3.0	1.0	<0.001	1.7 ± 1.4	0.214	0.0137	0.8 ± 0.7	1.0	0.0818	1.7 ± 0.8	0.00482	0.92278	6.6 ± 3.5	1.0	0.982	0.8 ± 0.7	0.0619	<1e−05	0.0 ± 0.0	0.9756	0.0536

Notes: ^a^The preference percentage of the media where destination equaled source was used as a reference for comparing the means of each test media. ^b^The mean preference percentage of the NGM destination was used as a reference for comparing the means of each test media. Ref indicates reference media for comparison.

### Isolation of wild nematodes on OP50-seeded NcM

While we have shown that laboratory acclimated *C. elegans* and *C. tropicalis* can grow well on three NcMs, a better test would be to isolate worms from the wild. We focused on the three best NcMs for *C. elegans* brood size (NcM8, NcM12, and NcM18) and NcM6. We went to Cat Tien National Park, collected 16 samples of rotting vegetation (leaf litter), and then plated the samples on *E. coli* OP50-seeded plates of these four NcMs and NGM. We observed nematodes from all 16 samples within seven days of plating on at least one of the media types, and then conducted additional analysis for one sample that was plated onto NcM18 (see Methods).

From this sample, we observed at least two different nematode species. Of these, we then picked two adult nematodes (isofemales), which appeared similar in size to *C. elegans,* onto separate OP50-seeded NcM18 plates to establish lines. We then singling out three L2 stage larvae for both strains and found that both strains were hermaphroditic (or possibly parthenogenetic). One strain, CFB206, had two circular pharyngeal bulbs suggesting that it might be *Caenorhabditis*. The other had only one circular posterior bulb while the anterior bulb was long and tapered toward the mouth; this strain was not pursued further. To classify CFB206 to the species level, the 18 S rDNA of this strain was amplified by PCR and sequenced (Floyd et al., 2002). A BLASTN homology searched revealed that CFB206 was *C. briggsae*. These results demonstrate that NcM18, and probably the other two “best” NcMs (NcM8 and NcM12) and NcM6 could be used as media in the isolation of nematodes, including *Caenorhabditis* species.

### *Growth of* C. elegans *on* Acinetobacter *sp. CFBb9-seeded NcM media*

*E. coli* OP50 is not a natural (Brooks et al., 2009) nor preferred food of wild *Caenorhabditis* species (Shtonda and Avery, 2006). Thus, we examined if NcMs could be used to culture natural bacteria associated with *Caenorhabditis*. To this end, we used the same sample containing *C. briggsae* and plated 100 μL of a 1,000-fold dilution of the sample onto an LB agar media plate. Greater than 100 colonies were observed after two days of growth. Of these, we chose to culture one colony, which was among the biggest, for DNA isolation. This strain, CFBb9, was identified as an isolate of *Acinetobacter* sp., a Gram-negative bacterium.

**Figure 5:**
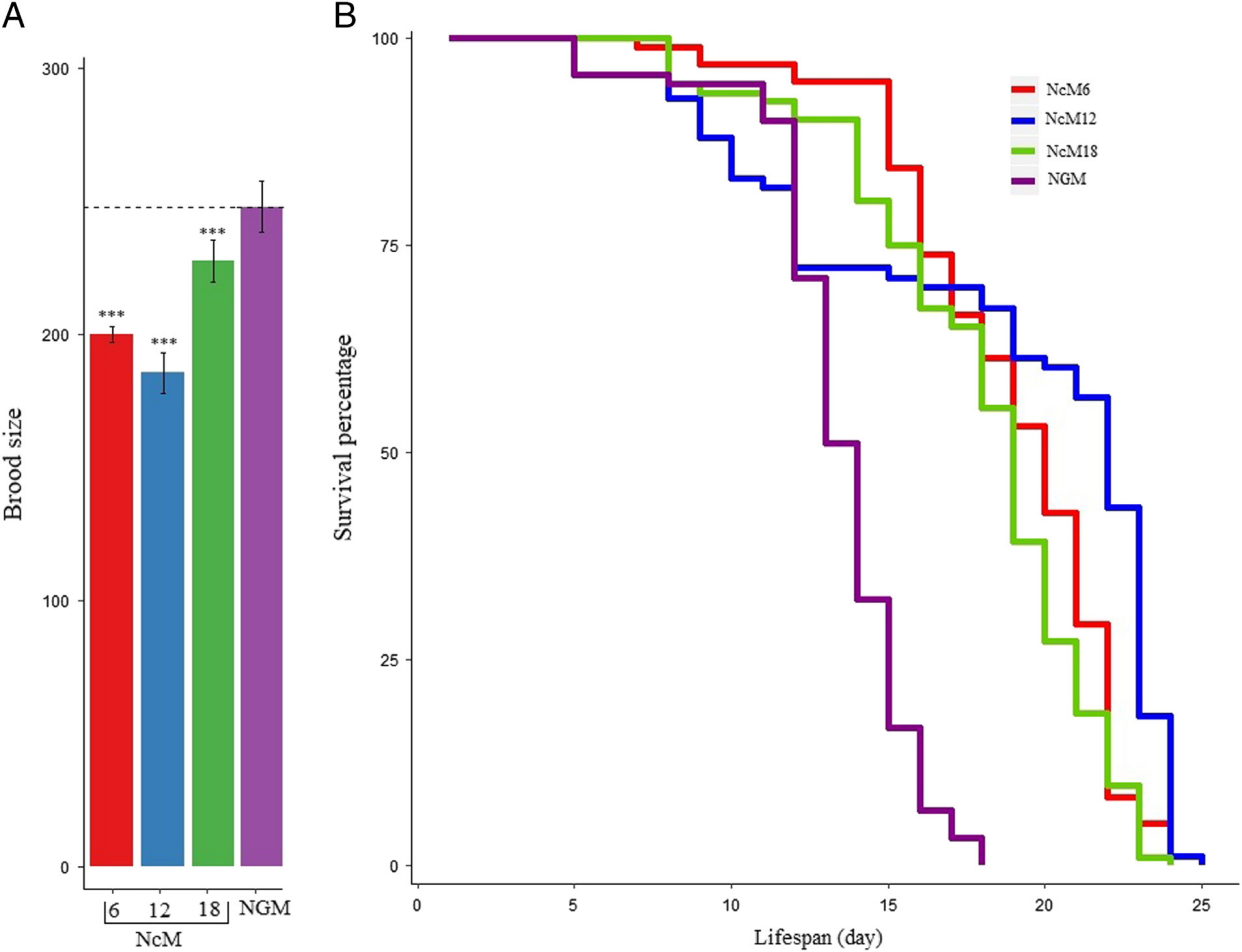
Growth assays of *C. elegans* on a natural bacterial strain, *Acinetobacter* sp. CFBb9. (A) The total brood sizes of worms differed among the four different types of media (*P* <* *2e−16, F** = **175.8, one-way ANOVA test), with that of worms growing on NcM6, NcM12, or NcM18 being lower than on NGM (***P < **0.001, Dunnett’s post-hoc test), see Table S9 for exact *P*-values. The standard errors (SE) and error bars for each data set are the SE among P0 batches and not the SE across individual P0s. (B) Worms grown on NcM6, NcM12, and NcM18 have longer lifespans than on NGM (all *P* < **0.001, log-rank test), see Table S10 for exact *P*-values.

We next characterized how *C. elegans* grows on CFBb9. The brood sizes of *C. elegans* on CFBb9-seeded NcM6, NcM12, and NcM18 were all lower (all means ≤ 227.7 ± 7.8) than that on NGM (250.1 mean ± 3.0 SE; all *P* < 1e−10; one-way ANOVA followed by Dunnett’s test for difference from NGM; Fig. 5A and Table S9). The lifespans on these three NcMs (all means ≥ 17.93 ± 0.41) were significantly longer than that on NGM (13.41 mean ± 0.26 standard error; all *P-*values < 2e−16, log-rank test; Fig. 5B and Tables S10 and S11).

**Table S9.** Brood size of *C. elegans* on *Acinetobacter* sp. CFBb9.

Media	Number of tested hermaphrodites	Total brood size (Mean ± SE)	Dunnett’s post hoc *P-*values
NGM	29	248.0 ± 9.7	Control
NcM6	38	200.2 ± 2.9	<1e−10
NcM12	39	185.6 ± 7.7	<1e−10
NcM18	38	227.7 ± 7.8	<1e−10

Note: Yellow shading highlights the NGM control. Brood sizes were lower on NcM plates. The standard errors (SE) and error bars for each data set are the SE among P0 batches and not the SE across individual P0s.

**Table S10.** Lifespan of *C. elegans* on *Acinetobacter* sp. CFBb9.

Media	Number of tested hermaphrodites	Lifespan (days) Mean ± SE	Log-rank *P-*value
NGM	90	13.41 ± 0.26	Control
NcM6	96	18.97 ± 0.35	<2e−16
NcM12	83	18.81 ± 0.63	9.4e−16
NcM18	92	17.93 ± 0.41	<2e−16

Note: Yellow shading highlights the NGM control. Lifespans were longer on NcM plates.

**Table S11.** Number of surviving *C. elegans* worms on *Acinetobacter* sp. CFBb9 at each day of the lifespan assays.

	Number of surviving worms			
Day	NGM	NcM6	NcM12	NcM18
1	90	96	83	92
2	90	96	83	92
3	90	96	83	92
4	90	96	83	92
5	86	96	83	92
6	86	96	83	92
7	86	95	83	92
8	85	95	77	87
9	85	93	73	86
10	85	93	69	86
11	81	93	68	85
12	64	91	60	83
13	46	91	60	83
14	29	91	60	74
15	15	81	59	69
16	6	71	58	62
17	3	64	58	60
18	0	59	56	51
19		51	51	36
20		41	50	25
21		28	47	17
22		8	36	9
23		5	15	1
24		0	1	0
25			0	

### Costs of NcMs

To determine the amount saved when using NcMs compared to NGM, we calculated the cost per liter of media, which can make approximately one hundred 6-cm plates, for all NcMs (Table 2). In Vietnam, the media costs of the three best NcMs (NcM8, NcM12, and NcM18) and NcM6 were $1.70 to $1.87 US per liter, which was approximately two-third of the cost for NGM ($2.00 US).

**Table 2.** Approximate cost of reagents for making 1 L of media or approximately one hundred 6-cm plates.

	Cost^a^ (VND)				
Components (amount/L)	NcM6	NcM8	NcM12	NcM18	NGM
Cholesterol (1 mL of 5 mg/mL)					200
Plant cooking oil (100 µL)	2				
Chicken egg (0.56 g)		56	56		
Pig fat (0.4 g)				28	
Autoclaved mushroom (20 mL)	115	115	115	115	
Peptone (2.5 g)					3000
Pure mineral mixture (1 mL of 1 M CaCl_2_, 1 mL of 1 M MgSO_4_, 25 mL of 1 M KPO_4_)		3788			3788
Soil solution (10 mL)	0		0	0	
Agar (17 g)	34,000	34,000	34,000	34,000	34,000
NaCl (4 mL of 0.75 g/ mL)	3,000	3,000	3,000	3,000	3,000
Distilled water with reverse osmosis (1 L)	2,000	2,000	2,000	2,000	2,000
Total cost in VND	39,117	42,959	39,171	39,143	45,988
Total cost in US$^b^	1.70	1.87	1.70	1.70	2.00

Notes: ^a^Cost in Vietnam Dong (VND) based on current prices on May 31, 2020 in Hanoi, Vietnam. ^b^Based on the exchange rate (23,000 VND to $1 US on May 31, 2020.

## Discussion

We have tested 25 new NcMs for rearing *C. elegans*. The main goal was to substitute the defined, but more expensive, components of NGM (cholesterol, minerals, and peptone) with common, less expensive materials that could be obtained from the local Vietnamese market, and in general, globally. Three of these NcMs (NcM8, NcM12, and NcM18) supported growth, as measured by brood size and lifespan, that was not different from NGM (Tables S1, S3 and S4; Figss 2A and 3A). A fourth NcM, NcM6, also performed well, being only a little inferior to NGM.

In general, the success of the three best NcMs and NcM6 makes sense based on their composition compared to that of the unsuccessful NcMs. Each of the four had presumptive replacements for cholesterol (egg, pig fat, or mushroom), minerals (soil, except NcM8 which had the standard worm mineral mixture), and peptone (mushrooms). The other NcMs were missing one or more of these components. Based on a report that *C. elegans* grows well on cultivated mushroom *Agricus bisporus* (Grewal and Richardson, 1991), we had predicted that the mixture of straw mushrooms (*V. volvacea*) and oyster mushrooms (*P. ostreatus*) plus a mineral source could support worm culturing. However, this was not the case as worm growth was inferior on NcMs with mushrooms but lacking a cholesterol source compared to those with a cholesterol replacement.

The NcM components are not completely defined, and thus, there are two issues. First, in addition to the intended replacement elements, there are likely other unknown elements. For example, our sample soil was associated with vegetation, so it likely had organic compounds from plants, animals, and living microorganisms (Flaig, 1971). These compounds could provide more nutritional factors for both OP50 and *C. elegans*. On the other hand, soil samples may contain toxic reagents such as heavy metals for *C. elegans* (Jonker et al., 2004). Because there was robust grass growth above our soil sample, our soil sample presumably had low levels of toxic elements. Similarly, whole eggs are quite nutritious and likely provides a rich source of additional protein and vitamins. Second, because the raw materials could be slightly different at different geographic locations, there may be variation in the performance of the three best NcMs (NcM8, NcM12, and NcM18), possibly requiring location-specific minor recipe adjustments.

### *OP50-seeded NcMs as general media substitutes for* Caenorhabditis *species*

While *C. elegans* N2 is called “wildtype”, it is really a domesticated laboratory strain (Sterken et al., 2015), selected for growth on OP50-seeded NGM. Good growth of N2 on the three best NcMs and NcM6 may not reflect good growth of other *Caenorhabditis* species on these media. Rather these media may have been inadvertently tailored for *C. elegans* or are good mimics for laboratory conditions. We conducted two experiments to examine if some of the four NcMs could be general substitutes for growing *Caenorhabditis* species.

First, we found that *C. tropicalis* BRC20400 grew well on NcM12 and NcM18 (two of the three best; NcM8 was not tested) and NcM6 (Figs. 2B and 3B; Tables S2 and S5). Furthermore, *C. tropicalis* had larger brood sizes on these NcMs than on NGM. This result implies that NGM may have less or is missing some nutritional components for optimal growth of *C. tropicalis*. While every species is different, this result further suggests that testing difficult-to-grow (on NGM) nematode species on different media may be worthwhile with NcM6, NcM8, NcM12, or NcM18 as possible good starting points.

Second, we were able to isolate and raise a wild strain of *C. briggsae* found in leaf litter on NcM18. As noted above, *C. tropicalis* BRC20400, which is a strain recently isolated from Taiwan and has had only a brief time to evolve under lab conditions, also grew well on NcM18. Thus, NcM18 can be used to grow at least three species including both lab acclimated strains (*C. elegans* N2) and wild (or nearly so) strains (*C. briggsae* and *C. tropicalis*). Given (1) that OP50-seeded NGM has been used to isolate and raise many wild *Caenorhabditis* species, (2) the three best NcMs (NcM8, NcM12, and Ncm18) and NcM6 are good media for *C. elegans*, (3) and the NcM18 results, we suggest by extension that all of these four NcMs would also be viable options to isolate and raise many other wild *Caenorhabditis* species.

### Lack of media preference

In many insects, the adults seem to prefer the host species (usually plant) on which they grew up as larvae (Davis and Stamps, 2004). Related, prior studies have shown that when adult *C. elegans* are given a choice, they migrate to the temperature experienced during their growth (Hawk et al., 2018; Hedgecock and Russell, 1975). Using our different NcMs, we tested if *C. elegans* preferentially migrated to the destination media that matched the source media. However, we found no relationship (Fig. 4; Tables S7 and S8). Thus, at least within the limits of our experimental design (e.g., only 2 h for searching), prior experience (source) was not very strong. Nevertheless, there may be a hint that source experience (possibly coupled to aversion to some NcMs, including the source NcM) may affect choices because destination preference patterns were different when the source media was NcM9 or NcM12 (at least when compared simultaneously to the five other NcMs; Fig. 4; Tables S7 and S8). Additional studies would be required to better understand growth experience on adult media preference.

### *Growth of* C. elegans *on NcMs seeded with* Acinetobacter *sp. CFBb9*

*C. elegans* is most commonly fed *E. coli* OP50 in the laboratory, but *C. elegans* rarely encounters *E. coli* in the wild. Studying the natural food that *C. elegans* or other *Caenorhabditis* species eat would be interesting (Darby, 2005; Maynard and Weinkove, 2020; Meisel and Kim, 2014; Poupet et al., 2020). We tested and found that NcM6, NcM12, and NcM18 could support the growth of a natural bacterial isolate, an *Acinetobacter* sp. strain CFBb9, associated with a sample containing *C. briggsae*. Furthermore, CFBb9 could serve as food for *C. elegans*. We found that the worms feeding on CFBb9-seeded NcM plates had smaller brood sizes and greater longevity than those feeding on CFBb9-seeded NGM plates (Fig. 3 and Table S9 and S10). This is the opposite pattern of that found for OP50-seeded plates. Thus, the media type and food can interact to affect *C. elegans* physiology, as has been found in previous studies (Yu et al., 2015). Finally, the ability of both *E. coli* and *Acinetobacter* sp. to grow on these NcM plates suggests that isolating other natural bacteria directly onto these NcM plates is possible. Overall, this implies that surveying of natural bacteria concomitantly with wild *Caenorhabditis* is possible with these NcM plates.

### Cost

NcMs were cheaper than NGM but depending on local markets, the cost reduction will obviously vary. In Vietnam, the cost of 1 L of NGM, which makes approximately one hundred 6-cm plates, is about $2.00 US (Table 2). The costs of 1 L of NcM6, NcM8, NcM12, and NcM18 range from $1.70–1.87 US, which is equivalent to a savings of 6.5–15% ($0.13–$0.30 US; Table 2). These four NcMs notably lacks peptone, which is one of the more expensive reagents in Vietnam. If disposable plastic Petri plates are used ($17–26 US for 100 plates), then the savings for 100 plates would be modest, 0.5–1.6%. This cost can be reduced if reusable glass Petri plates are used, as we did in this series of experiments. Glass Petri plates are an expensive one-time initial setup cost ($100–130 US per 100 plates), but subsequent usage is the price of washing, which we estimate to be about $3.00 US (for 100 plates; labor, water, and electricity in Vietnam). In this case and ignoring the setup cost, the financial savings is still appreciable, 2.6–6.0%. The prices of hardware and labor also vary globally, therefore each lab’s real savings may differ from our calculation. Nevertheless, NcMs should always be cheaper than NGM.

A related issue is the availability of the components, especially the mushrooms. While oyster mushrooms seem more global, fresh straw mushrooms may be harder to find outside of Asia. A financially equivalent mushroom (or reagent) will need to be identified in such countries.

## Conclusions

We have developed three cheaper NcM recipes that can replace NGM. The components of these NcMs can be obtained in many places. Nevertheless, eggs should be the easiest to find in markets everywhere, so we recommend NcM12 as it has the fewest components and is the cheapest for researchers with limited budgets who study *Caenorhabditis* and other nematode species.
